# Pain, Physical Demands at Work, and Future Work Expectations Among Older Adults in the United States

**DOI:** 10.1093/geroni/igad089

**Published:** 2023-08-22

**Authors:** Theresa Andrasfay, Gillian Fennell, Eileen Crimmins

**Affiliations:** Department of Public Health, California State University San Marcos, San Marcos, California, USA; Leonard Davis School of Gerontology, University of Southern California, Los Angeles, California, USA; Leonard Davis School of Gerontology, University of Southern California, Los Angeles, California, USA

**Keywords:** Disability, Retirement, Working longer

## Abstract

**Background and Objectives:**

In the United States, pain is becoming increasingly prevalent among older adults at the same time as policies are incentivizing work longer. Given that pain and physically demanding jobs are both linked to early retirement and they often go hand-in-hand, it is important to assess how the unique effects of pain and physical work demands may interact in predicting future work expectations.

**Research Design and Methods:**

Using Health and Retirement Study data (1998, 2004, 2010, and 2016 waves), we assess how pain and physical job demands influence future work expectations of 10,358 adults at midlife (ages 51–56), after accounting for sociodemographic, job, health, and financial characteristics.

**Results:**

Compared to men with no pain, activity-interfering pain was associated with low expectations of full-time work past 62 regardless of job demands, while noninterfering pain was associated with 62% higher odds (odds ratio [OR] = 1.62, 95% confidence interval [CI]: 1.35–1.93) of expecting not to work full-time past age 62 only among those with physically demanding jobs. Having both interfering pain and a physically demanding job was associated with increased odds of expecting not to work full-time past age 65 for men (OR = 1.25, 95% CI: 1.06–1.47) and past age 62 for women (OR = 1.18, 95% CI: 1.00–1.39).

**Discussion and Implications:**

The co-occurrence of physically demanding work with pain—particularly activity-interfering pain—is associated with low expectations of full-time work past ages 62 and 65 for adults at midlife. Working longer may be feasible for older adults whose pain does not interfere with work, but unrealistic for individuals facing both pain and physically demanding work.

**Translational Significance:** Experienced pain and physical work demands independently predict labor force participation, but it is unclear how their co-occurrence influences future work expectations. We examined associations between pain interference and low expectations for full-time work past ages 62 or 65 by occupational physical demands. In general, individuals experiencing both interfering pain and highly physically demanding jobs were particularly likely to report low expectations of working past these ages. As the prevalence of pain increases among adults nearing retirement age, employers and policy-makers should consider that the intersection between pain and work demands may limit individuals’ ability and desire to work longer.

Pain is a prevalent and growing public health concern in the United States, affecting approximately 20% of Americans (Zajacova et al., 2021a; Zelaya et al., 2020). Among those with elevated risk for developing pain are individuals working physically demanding jobs (Hagen et al., 2002; Osinuga et al., 2021; Stoesz et al., 2020). Such jobs are characterized by frequent lifting, stooping, and carrying, and while these tasks are most frequently associated with working in construction, foresting/fishing, or cleaning jobs, they can occur in numerous occupations and are not uncommon among older workers (Andrasfay et al., 2021; California Department of Industrial Relations, 1999).

Both pain and working physically demanding jobs are individually associated with a higher likelihood of early retirement (Gignac et al., 2019; Salonen et al., 2018; Scott et al., 2018; Welch et al., 2010). Pain often limits mobility and physical functioning (Grabovac & Dorner, 2019; Zimmer & Rubin, 2020), and the ability to do physically demanding work requires strength and stamina, both of which decrease with age (Keller & Engelhardt, 2014). Physically demanding work can also exacerbate pain among those already experiencing pain, which may lead workers to perceive continued strenuous work as a threat to their quality of life (Kalichman & Hunter, 2007; National Academies of Sciences et al., 2019; Petersen et al., 2019; Sundstrup & Andersen, 2017).

Social Security disability insurance (SSDI) provides benefits to individuals unable to work due to a medical condition (Social Security Administration, 2022a). However, SSDI applicants are required to present a specific medical diagnosis as proof of their inability to work, and this requirement disqualifies many older adults with pain as its causes are often unknown or not medically verifiable (Houtenville & Ozabaci, 2019; Maestas, 2019; Zajacova et al., 2021b). Individuals who cannot continue working due to pain but do not qualify for SSDI benefits may instead choose to claim early retirement benefits. Because claiming benefits before one’s full retirement age translates to lower monthly Social Security payments—and this penalty increases for cohorts with higher full retirement ages—claiming early retirement benefits can leave Americans who primarily rely on Social Security benefits with insufficient income in retirement (Gassoumis et al., 2011; Li, 2022). This may be particularly true for workers in physically demanding jobs, as workers in service and manual occupations are paid below the national average income and are less likely to have access to employer-sponsored retirement benefits (Bui, 2014; Clougherty et al., 2010; Schofield et al., 2011; U.S. Bureau of Labor Statistics, 2022). Prior research also finds that the prevalence of pain is higher among individuals with lower education and income levels (Case et al., 2020; Zajacova et al., 2021a). Because individuals in pain and/or working physically demanding jobs are, on average, more socioeconomically disadvantaged than the total population, early retirement should be considered a major financial risk for individuals with pain, physical work demands, or both.

Given that experiencing pain and working physical jobs are both linked to early retirement *and* they so often co-occur, it is important to assess how the unique effects of pain and physical work demands may interact in predicting future work expectations of older adults in the United States. Although prior studies have investigated the consequences of the mismatch between workers’ physical abilities and the demands of their jobs (Fraade-Blanar et al., 2017; Hudomiet et al., 2018; Lopez Garcia et al., 2019; Sonnega et al., 2018), there are no known studies investigating the moderating effect of physical work demands on the relationship between pain and future work expectations. Understanding the unique and intersecting roles of both of these factors on future work expectations may provide insight into the feasibility of policies to incentivize working longer among individuals impacted by these factors (Baxter et al., 2021; Solem et al., 2016).

Evidence for the accuracy of future work expectations for predicting future labor force participation is mixed. Taking into account the normative age of retirement in their field, their current age, marital status, finances, health, and functional mobility, individuals have been found to forecast their retirement age fairly accurately (Hurd & Rohwedder, 2022; Ilmakunnas & Ilmakunnas, 2018). However, another study using HRS data reported varying levels of accuracy depending on respondents’ reported chances of working full-time after the target age (Abrams et al., 2022). For example, workers who reported very low probabilities of working past 62 were more accurate about their employment status at age 62 than those who reported very high probabilities of working past age 62, likely because unforeseen layoffs or emergent health shocks can prevent individuals from working as long as planned (Abrams et al., 2022). That said, regardless of the *accuracy* of future work expectations, this subjective metric provides meaningful insight into individuals’ perceived health, work ability, and financial preparedness for retirement (Baxter et al., 2021; Solem et al., 2016).

Using data from the Health and Retirement Study (HRS), we assess how pain and physical job demands influence the future work expectations of adults at midlife, an important time in the life course when many individuals are seriously considering their plans for retirement. We expect that respondents reporting pain-related interference with daily activities and current employment in a physically demanding job will be more likely to report that they do not expect to continue working full-time past age 62 and/or age 65 than individuals experiencing pain without these job demands. Here, we conceptualize expectations about full-time work past ages 62 and 65 as proxies for expected early and traditional retirement, as age 62 is the age of early eligibility for Social Security benefits and age 65 is when individuals become eligible for Medicare and was the original eligibility age for full Social Security benefits (Social Security Administration, 2022b). We conduct separate models for men and women to answer our research question for three reasons. First, physical work demands predict disability retirement more strongly for men (Emberland et al., 2017; Falkstedt et al., 2021). Second, women experience higher rates of activity-limiting pain (Stubbs et al., 2010). Third, associations between other covariates and future work expectations may vary by gender. For example, married women are more likely to retire at the same time as their husbands despite being, on average, several years younger, and marital status is more strongly related to financial preparedness to retire for women than men; thus the association between marital status and work expectations may vary by gender (Groneck & Wallenius, 2021; Ho & Raymo, 2009).

## Data

We conduct the present investigation using data from the 1998, 2004, 2010, and 2016 waves of the HRS. The HRS is sponsored by the National Institute on Aging (grant number NIA U01AG009740) and is conducted by the University of Michigan. The HRS is a longitudinal study (1992–2020) with a nationally representative sample of Americans aged 51 and older (Sonnega et al., 2014). We use data from the 1998 wave and beyond because the survey questions for the variables we use were not consistent across waves prior to that year. We use data from every third wave after 1998 because new cohorts of respondents (aged 51–56) were added to these waves. In these “refresher” cohort entry waves, all newly recruited respondents were interviewed using comprehensive questionnaires; in other waves, some questions were only asked of new interviewees or alternating halves of the sample. Data used in this study are obtained from the HRS Tracker File 2018, RAND HRS Longitudinal File 2018, and the RAND HRS Fat Files (Health and Retirement Study, 2022; RAND, 2013; RAND, 2018; RAND, 2019; RAND, 2021).

The age range of our sample was restricted to those aged 51–56 (i.e., the newly recruited respondents at these waves) who were working for pay, thus allowing our investigation of how their current working conditions are associated with future work expectations. We did not exclude individuals working part-time as descriptive analyses showed that part-time workers were more likely to report interfering pain relative to full-time workers. Also, approximately half of the part-time workers in our sample reported a greater than 25% chance of working full-time at the target ages. This suggests that part-time work at baseline may have been situational and not permanent, such that they were unable to find full-time work at the time or needed to temporarily work part-time to balance caregiving responsibilities. We excluded respondents whose interviews were completed by a proxy and whose responses to the questions about future work expectations were missing or inconsistent across these ages (i.e., reporting a higher probability of working past age 65 than 62), resulting in a final analytic sample composed of 10,358 unique respondents. This sample selection process is shown in Figure A1.

## Measures

### Future Work Expectations

Employed respondents were asked about the likelihood they would be working full-time past ages 62 and 65. Using these expectancy variables instead of a measure of actual retirement enables the investigation of individuals who are still several years away from retirement without having to wait for them to exit the labor force. The first question asked, “Thinking about work in general, and not just your present job, what do you think the chances are that you will be working full-time after you reach age 62?” Respondents were then asked about their chances of working full-time after age 65. Respondents provided the probability of working full-time, between 0% (completely unlikely) and 100% (very likely). We theorize that those who report very low probabilities have clear reasons for not wanting or not being able to work past these ages that are likely to be influenced by pain or strenuous job conditions, so for this analysis, responses are dichotomized into those with a very low expectation (0%–25% chance) that they would be working full-time and those with higher expectations (26%–100% chance). The distribution of the raw responses to these questions is shown in Figure A2. Although we focus on dichotomization at 25% for our main analyses, we also considered alternative cutpoints (0% and 50%) for this outcome in supplementary analyses.

### Pain Interference

HRS respondents were first asked if they were often troubled by pain (*yes*/*no*). If a respondent reported pain, they were then asked if they experience pain-related interference with daily activities (i.e., “household chores or work”). From these two questions, we created a three-category measure of pain with categories for no pain, noninterfering pain, and interfering pain. Although the HRS also asks questions about pain severity, we use measures of pain interference because previous research has shown that regardless of intensity, pain that does not interfere with daily activities has less influence on imagined futures due to its lesser impact on day-to-day life (Fennell et al., 2022). To test if our results were sensitive to this analytic decision, we also consider pain presence and pain intensity in supplementary analyses.

### Physical Demands at Work

Working respondents were asked the frequency with which their job requires lots of physical effort, with potential responses including all or almost all of the time, most of the time, some of the time, or none or almost none of the time. We dichotomized these response options so that respondents who indicated “all or almost all of the time” were coded as having a high physical effort job (1) and individuals in the other categories were coded as not having a high physical effort job (0), but results were similar if we included “most of the time” in the high physical effort category.

### Covariates

We include an extensive set of covariates, including sociodemographic, job, health, and financial characteristics related to labor force participation, retirement readiness, pain, and physical job demands.

#### Sociodemographic characteristics

We control for age, race/ethnicity (categorized as non-Hispanic White, non-Hispanic Black, Hispanic, and non-Hispanic other), foreign-born status, marital status (categorized as married/partnered, separated divorced, widowed, and never married), and educational attainment (categorized as a college degree or higher, some college, high school or equivalent, and less than high school).

#### Job-related measures

Job characteristics include the broad category of the respondent’s main current occupation (categorized as managerial/professional, sales/clerical, service, and manual), typical weekly work schedule (categorized as part-time if less than 35 hours per week, full-time if 35–54 hours per week, and more than full-time if 55 or more hours per week), as well as indicators for whether the respondent is self-employed, a member of a union, and strongly agrees that their current job is stressful.

#### Health

Health characteristics include smoking status (never smoker, former smoker, and current smoker), obesity (body mass index greater than or equal to 30 coded as 1), depression (positively endorsing 3 or more symptoms on the Center for Epidemiologic Studies Depression (CESD-8) scale), and the count of self-reported chronic conditions (including diabetes, hypertension, heart disease, cancer, and lung disease).

#### Financial measures

We control for the presence of a defined benefit retirement plan (e.g., traditional pension) at the respondent’s current job, as well as several financial variables at the household level: income, nonhousing net worth, debt, total individual retirement account (IRA) balances, and total defined contribution account balances (e.g., 401k). Household income includes employment earnings, pension or retirement benefits, unemployment or disability benefits, capital income, and other income. Household nonhousing net worth excludes balances held in IRAs to avoid double counting such assets (For individuals with a negative net worth on the above household nonhousing net wealth variable, we coded their net worth as $0 and have created a separate debt variable to capture the amount of debt). We have standardized all income and asset values to 1998 dollars to account for inflation; we also log-transformed these variables (adding $1 to each to include individuals with $0 of income or assets) for the analysis to adjust for skewed distributions. Additional details on these financial variables are shown in Appendix A3. In addition to these measures of household finances, we also include the total number of years worked and the age at which respondents will be eligible for full retirement benefits based on their birth cohort as these both influence the amount respondents can expect to receive in Social Security benefits if claiming at age 62 or 65.

## Analysis

To assess the associations between pain interference, physical demands at work, and expectations about future full-time work, we fit a series of logistic regressions. Model 1 uses pain interference and high physical demands at work to predict whether respondents expect to stop working full-time by age 62. Model 2 additionally includes the interaction between pain interference and high physical demands at work. Both models include all sociodemographic, employment, health, and financial characteristics as covariates. Models 3 and 4 are analogous to Models 1 and 2 but predict whether respondents expect not to work full-time past age 65. We stratified all analyses by gender to allow for these characteristics to differentially influence expectations about future work for men and women. Respondent sample weights are included in all analyses to account for the complex sampling structure of the HRS.

Approximately 16% of the analytic sample was missing values for at least one of the predictors or covariates of interest; the percentage of the sample missing data on each variable is shown in Table 1. To address this line-item missingness, we have created 10 multiply-imputed data sets using the Multivariate Imputation by Chained Equations (mice) package in R (van Buuren & Groothuis-Oudshoorn, 2011). To improve the quality of the imputations, we used all variables included in the analysis to estimate missing values as well as several additional variables, including a diagnosis of arthritis, pain severity, respondents’ longest occupation reported to the HRS, number of mobility limitations, and self-reported work limitation due to a health problem. We included the outcome variables as predictors for these imputations but dropped respondents missing the outcomes before analyzing the data. All results presented subsequently are pooled across the 10 imputations to account for the variability across the multiple imputations.

**Table 1.** Summary Statistics of Analytic Sample by Gender

Characteristic	Men (*n* = 4,691)		Women (*n* = 5,667)		Gender difference (*p* value)	% of sample missing data
	%	Mean (*SD*)	%	Mean (*SD*)		
Expectations for future work						
Expects not to work full-time past 62	25.3		34.9		***	0
Expects not to work full-time past 65	46.3		55.9		***	0
Pain interference with activities						<1
No pain	75.0		70.7		***	
Pain does not interfere with activities	12.9		12.4			
Pain interferes with activities	12.1		16.9		***	
High physical effort job	22.0		19.4		**	<1
Sociodemographic characteristics						
Age		53.5 (1.7)		53.5 (1.7)		0
Race/ethnicity						<1
Non-Hispanic White	76.0		76.4			
Non-Hispanic Black	8.6		10.4		*	
Hispanic	9.2		8.5			
Non-Hispanic other	6.2		4.7		*	
Foreign-born	12.8		9.7		***	<1
Marital status						<1
Married/partnered	78.1		69.4		***	
Separated/divorced	14.1		19.9		***	
Widowed	1.0		4.2		***	
Never married	6.8		6.6			
Educational Attainment						<1
College degree or higher	35.1		33.6			
Some college	12.8		13.6			
High school or equivalent	44.1		46.5		*	
Less than high school	8.0		6.3		***	
Employment characteristics						
Occupational category						1.0
Managerial/professional	38.5		41.9		***	
Sales/clerical	15.3		32.4		***	
Service	8.9		17.8		***	
Manual	37.3		7.9		***	
Self-employed	19.4		14.0		***	<1
Belongs to a union	19.4		17.3		**	<1
High stress	23.3		28.3		***	<1
Work hours						1.6
Part-time	9.7		25.3		***	
Full-time	72.3		66.5		***	
More than full-time (55+ h)	18.1		8.3		***	
Health characteristics						
Smoking status						<1
Never smoker	43.8		49.7		***	
Former smoker	35.1		31.9		***	
Current smoker	21.0		18.4		***	
Obese	33.2		35.5		*	1.9
Three or more depressive symptoms	13.5		18.8		***	<1
Count of chronic conditions		0.61 (0.77)		0.58 (0.76)	*	<1
Financial characteristics						
Household income and assets (1998 dollars)						
Income^a^		92,760 (148,000)		81,120 (122,600)	***	0
Nonhousing financial wealth^a^		86,830 (350,200)		77,020 (290,800)		0
Nonmortgage debt^a^		4,807 (40,340)		3,675 (17,130)		0
Defined contribution plan balance		47,780 (189,000)		40,490 (187,500)	*	8.9
IRA balance^a^		39,950 (130,100)		48,850 (317,700)	**	0
Benefits-eligibility characteristics						
Defined benefit plan at current job	30.5		28.8			2.0
Full retirement age		66.4 (0.5)		66.4 (0.4)		0
Total years worked		27.9 (10.3)		25.7 (9.9)	***	0

*Notes*: Data are from the Health and Retirement Study (HRS) from the 1998, 2004, 2010, and 2016 waves. The sample is restricted to individuals who were aged 51–56, were working for pay, and did not have a proxy respondent during these waves. IRA = Individual Retirement Account; *SD* = Standard Deviation.

^a^ These variables were previously imputed by RAND.

*p < .05; **p < .01; ***p < .001.

## Results

Summary statistics of the analytic sample are shown separately by gender in Table 1. Approximately one-quarter of men did not expect to work full-time past age 62, while over one-third (34.9%) of women did not expect to work full-time past this age. Nearly half (46.3%) of men in our sample did not expect to work full-time past age 65, while over 55% of women did not expect to work full-time past this age. With respect to pain, women reported a higher prevalence of any pain (29.3%) and pain-related interference (16.9%) with daily activities relative to men (25% and 12.1%, respectively). Men (22%) were slightly more likely than women (19.4%) to report having a physically demanding job, which may reflect their greater representation in the manual and service occupation categories (nearly half of men compared with a quarter of women).

Results from the models predicting the expectation of not working full-time past 62 are shown in Table 2 (the full set of coefficients is shown in Appendix A4). Model 1 found that, relative to men with no pain, men with interfering pain had 19% higher odds (OR = 1.19, 95% CI: 1.09 to 1.30) of expecting not to work full-time past age 62. In the same model, men with physically demanding jobs had 19% higher odds (OR = 1.19, 95% CI: 1.10 to 1.27) of expecting not to work full-time past age 62 compared to men whose jobs are not considered physically demanding. Model 2 added the interaction term between pain interference and physically demanding work to Model 1. This model indicates that the association between noninterfering pain and future work expectations depends upon physical job demands: among men without physically demanding jobs, noninterfering pain was not significantly associated with expecting not to work full-time past age 62, but among men whose jobs require high physical effort, having noninterfering pain was associated with 62% higher odds of expecting not to work full-time past age 62 compared to men without pain (OR = 1.62, 95% CI: 1.35 to 1.93). In contrast, interfering pain is associated with 20% higher odds (OR = 1.20, 95% CI: 1.08 to 1.33) of expecting not to work full-time past age 62, and this association does not depend on physical job demands. The main effect for a high physical effort job indicates that for those without pain, having a high physical effort job is associated with 10% higher odds (OR = 1.10, 95% CI: 1.01 to 1.19) of expecting not to work full-time past age 62.

**Table 2.** Associations Between Pain, High Physical Effort Work, and Expectation of not Working Full-Time Past Age 62: Odds Ratios and 95% Confidence Intervals

Variable	Men		Women	
	Model 1	Model 2	Model 1	Model 2
Pain interference (ref = no pain)				
Noninterfering pain	1.08 (0.99, 1.17)	0.93 (0.84, 1.03)	1.02 (0.95, 1.11)	**1.12** (1.02, 1.22)
Interfering pain	**1.19** (1.09, 1.30)	**1.20** (1.08, 1.33)	**1.21** (1.13, 1.30)	**1.16** (1.07, 1.25)
High physical effort job	**1.19** (1.10, 1.27)	**1.10** (1.01, 1.19)	**1.13** (1.06, 1.22)	**1.15** (1.06, 1.25)
High physical effort job X noninterfering pain		**1.62** (1.35, 1.93)		**0.69** (0.56, 0.85)
High physical effort job X interfering pain		1.01 (0.84, 1.21)		1.18 (1.00, 1.39)
Number of respondents	4,691	4,691	5,667	5,667

*Note*: Bold values indicate significance at *p* < .05. Results are pooled across 10 multiply imputed data sets. Models 1 and 2 both control for age, race/ethnicity, foreign-born status, marital status, educational attainment, main occupation, self-employment, union membership, job stress, work schedule, smoking status, obesity, depression, number of chronic conditions, possession of a defined benefit retirement plan, log household income, log household nonhousing net worth, log household debt, log household IRA balances, log household defined contribution account balances, total number of years worked, and the age at which respondents will become eligible for full retirement benefits. IRA = Individual Retirement Account.

**Figure 1.**
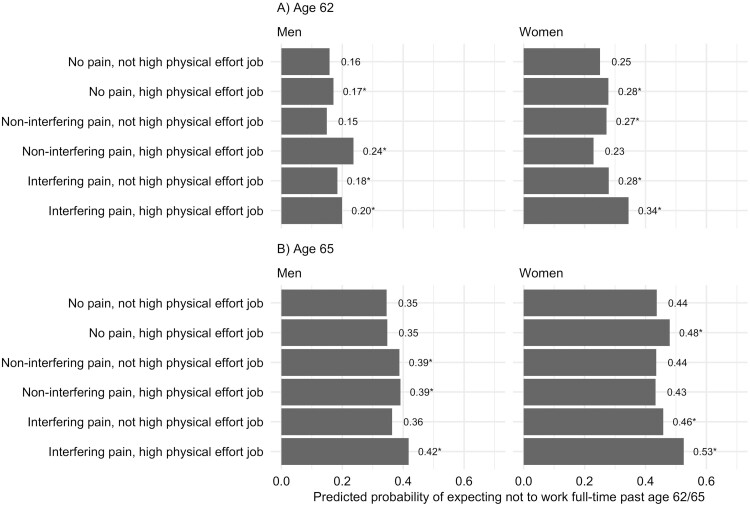
Predicted probabilities of expecting not to work full-time past (A) age 62 and (B) age 65 for men and women by level of pain interference and physical job demands. These predictions are from Model 2 (age 62) and Model 4 (age 65) and are calculated for a representative individual with the modal category for each of the categorical variables and mean levels of all numeric variables, with the exception of financial variables, which are set at the median. *Refers to a statistically significant difference (*p* < .05) relative to individuals with no pain and not high physical effort job.

The right half of Table 2 displays the results for women’s expectations of full-time work past age 62. Model 1 finds that, for women, both a high physical effort job (OR = 1.13, 95% CI: 1.06 to 1.22) and interfering pain (OR = 1.21, 95% CI: 1.13 to 1.30) are significantly associated with future work expectations, while noninterfering pain is not. In Model 2, the main effect of a high physical effort job remains significant, indicating that for women without pain, a high physical effort job is associated with 15% higher odds (OR = 1.15, 95% CI: 1.06 to 1.25) of expecting not to work full-time past age 62. For women without high physical effort jobs, noninterfering pain is associated with 12% higher odds (OR = 1.12, 95% CI: 1.02 to 1.22) and interfering pain with 16% higher odds (OR = 1.16, 95% CI: 1.07 to 1.25) of expecting not to work full-time past 62 compared to women without any pain in similar working conditions. The interaction term between high physical effort and interfering pain suggests that interfering pain may have a stronger association with work expectations at age 62 for women with physically demanding jobs, although this interaction does not reach statistical significance (OR = 1.18, 95% CI: 1.00 to 1.39). This model also finds an unexpected *negative* interaction between noninterfering pain and a high physical effort job, suggesting that among women in physically demanding jobs, those with noninterfering pain have 31% lower odds (OR = 0.69, 95% CI: 0.56 to 0.85) of expecting not to work full-time past age 62 compared to those without pain; as shown in Figure 1, this negative interaction serves to offset the main effects of noninterfering pain and high physical effort jobs, such that women with both noninterfering pain and high physical effort jobs have similar expectations about work at age 62 as women without pain in nonphysically demanding jobs

Table 3 shows the set of results from the models predicting the expectation of not working full-time past age 65 (the full set of coefficients is shown in Appendix A5). Among men, Model 3 finds that noninterfering pain is associated with 20% higher odds (OR = 1.20, 95% CI: 1.11 to 1.29) while interfering pain is associated with 15% higher odds (OR = 1.15, 95% CI: 1.06 to 1.25) of expecting not to work full-time past age 65 compared to men without any pain. In Model 4, the main effect of noninterfering pain remains significant (OR = 1.20, 95% CI: 1.10 to 1.31), but its interaction with high physical effort work is not, indicating that noninterfering pain is similarly associated with work expectations past age 65 for men with and without high physical effort jobs. In this model, the association between interfering pain and work expectations is significant only among men whose jobs required high physical effort (OR = 1.25, 95% CI: 1.06 to 1.47). Among women, Model 3 finds interfering pain to be associated with 12% higher odds (OR = 1.12, 95% CI: 1.05 to 1.20) and high physical effort work to be associated with 18% higher odds (OR = 1.18, 95% CI: 1.11 to 1.26) of expecting not to work full-time past age 65. In Model 4, both of these main effects remain significant, with interfering pain being associated with 9% higher odds (OR = 1.09, 95% CI: 1.01 to 1.18) and a high physical effort job associated with 19% higher odds (OR = 1.19, 95% CI: 1.09 to 1.29) of expecting not to work full-time past age 65. Interactions between pain interference and physical demands are not significant for women’s expectations of work past age 65.

**Table 3.** Associations Between Pain, High Physical Effort Work, and Expectation of not Working Full-Time Past Age 65: Odds Ratios and 95% Confidence Intervals

Variable	Men		Women	
	Model 3	Model 4	Model 3	Model 4
Pain interference (ref = no pain)				
Noninterfering pain	**1.20** (1.11, 1.29)	**1.20** (1.10, 1.31)	0.95 (0.88, 1.03)	0.99 (0.91, 1.08)
Interfering pain	**1.15** (1.06, 1.25)	1.08 (0.99, 1.18)	**1.12** (1.05, 1.20)	**1.09** (1.01, 1.18)
High physical effort job	1.05 (0.98, 1.12)	1.01 (0.93, 1.09)	**1.18** (1.11, 1.26)	**1.19** (1.09, 1.29)
High physical effort job X noninterfering pain		1.01 (0.85, 1.18)		0.83 (0.68, 1.03)
High physical effort job X interfering pain		**1.25** (1.06, 1.47)		1.10 (0.93, 1.31)
Number of respondents	4,691	4,691	5,667	5,667

*Note*: Bold values indicate significance at *p* < .05. Results are pooled across 10 multiply imputed data sets. Models 3 and 4 both control for age, race/ethnicity, foreign-born status, marital status, educational attainment, main occupation, self-employment, union membership, job stress, work schedule, smoking status, obesity, depression, number of chronic conditions, possession of a defined benefit retirement plan, log household income, log household nonhousing net worth, log household debt, log household IRA balances, log household defined contribution account balances, total number of years worked, and the age at which respondents will become eligible for full retirement benefits. IRA = Individual Retirement Account.

To facilitate the interpretation of these results, Figure 1 shows the predicted probabilities of expecting not to work full-time past ages 62 and 65 by gender, degree of pain interference, and presence of physical job demands. This figure is based on the results of Models 2 and 4, which included the interactions between pain interference and physical job demands. The displayed predictions are for a representative individual whose covariates are set at the sample mean values or modal category of all covariates, with the exception of financial variables, which are set at the sample medians. This figure shows that individuals reporting both interfering pain and a high physical effort job typically have the highest probability of expecting not to work full-time past age 62 or 65. The only exception is that men reporting noninterfering pain and a high physical effort job have the highest probability of expecting not to work full-time past age 62.

## Supplementary Analyses

Although our main specification defined self-reported probabilities of 25% or less as low expectations for full-time work past ages 62 and 65, we also considered a stricter cutoff of 0% and a more inclusive cutoff of 50% or less in supplementary analyses (Appendix A6). These new model specifications yield slightly different results with changes in effect magnitudes, and in some cases, changes in significance. The most notable difference is that with the 50% cutoff, we find that noninterfering pain is positively associated with expecting not to work full-time past age 62 for women in physically demanding jobs. This comes in contrast to the unexpected negative interaction between noninterfering pain and a high physical effort job in the main results. In general, even with these differences, the conclusions from these models remain the same: individuals with pain are more likely to report low expectations of full-time work past traditional retirement ages.

In addition to the main analysis based on pain interference, we also considered two alternative measures of pain, including a binary measure of any pain compared to no pain, and a measure of pain severity (no pain, mild, and moderate/severe pain). In the models based on the binary measure of pain (Appendix A7), pain was a significant predictor of future work expectations at 62 for both men and women, but the interaction between pain and physically demanding work was significant only for men’s expectation to not work full-time past age 62. This binary measure was significantly associated with work expectations past age 65 only for men. In the models based on pain severity (Appendix A8), the results for mild pain were similar to those for noninterfering pain in the main analyses whereas the results for moderate/severe pain were similar to those for interfering pain in both direction and magnitude, although not as consistently significant. These suggest that experiencing disruptive pain, whether due to severity or interference with activities, is associated with reduced expectations of future full-time work among those with physically demanding jobs.

## Discussion

Pain and physical work demands have been identified as independent predictors of early retirement in prior work (Gignac et al., 2019; Haukenes et al., 2011; Leinonen et al., 2011; Schreurs et al., 2011). In the present study, we examined the interaction effect of these two factors on the odds of expecting not to work full-time past ages 62 and 65 among middle-aged Americans in the HRS to improve our understanding of how pain and physical demands influence individuals’ plans for future work. In other words, we assessed whether pain (that does or does not limit daily activities) is more strongly associated with a greater perceived likelihood of no longer working full-time by 62 or 65 among men and women with physically demanding jobs. We expected that pain that interferes with daily activities would be more strongly associated with future work expectations for individuals with physically demanding jobs than for those without. This expectation was driven by prior work showing that persistent pain is incompatible with long working lives and that strenuous jobs exacerbate pain.

Although our findings do partially support our hypotheses, the story is not as clear as expected—especially for men. We expected that interfering pain would be a stronger predictor of not expecting to work full-time past age 62 for men with physically demanding occupations, but the association was similar for all men regardless of job demands. This could reflect the fact that pain can interfere with one’s ability to do other tasks at work besides those traditionally considered to be physically demanding. For example, low back pain and carpal tunnel pain can interfere with work requiring prolonged sitting and typing in a similar way to how musculoskeletal pain interferes with jobs requiring prolonged standing or heavy lifting. Interestingly, it was only among men with physically demanding jobs that noninterfering pain was associated with higher odds of expecting not to work full-time past age 62. This could indicate that men may only perceive nonlimiting pain as an impediment to full-time work past age 62 if the job is physically demanding.

The opposite associations were found when estimating the odds of expecting not to work full-time past age 65 for men. Activity-limiting pain was positively associated with this expectation, but only for men with physically demanding jobs. On the other hand, noninterfering pain was associated with men’s expectations of full-time work at age 65 regardless of physical job demands. It is possible that these men reporting noninterfering pain in their early to mid-50s expect their pain to worsen by the time they are 65, such that even if their pain does not currently limit their activities, it may limit their activities in the future.

The findings for interfering pain among women are more in line with our hypotheses. In our interaction models, interfering pain was associated with increased odds of expecting not to work full-time past age 62, especially for women with physically demanding jobs, though not significantly so. However, the finding that noninterfering pain was associated with *lower* odds of expecting not to work full-time past age 62 among women with physically demanding jobs was unexpected. It is possible that women who have both noninterfering pain and physically demanding jobs are disadvantaged in ways we are not adequately capturing with our financial measures that make working full-time past age 62 a financial necessity. In supplementary analyses in which we included more uncertain expectations in our definition of low expectations of full-time work past age 62, we found that noninterfering pain was associated with *higher* odds of expecting not to work full-time past age 62 among women with physically demanding jobs. This suggests that whereas individuals with physically demanding jobs are less likely to report very low probabilities of full-time work past age 62, they are more likely to express some uncertainty about working full-time past 62. When examining women’s expectations about full-time work past age 65, we found that both interfering pain and physically demanding work were independent predictors of expecting not to work full-time past this age, but there was no additional effect from having both of these factors.

Despite the incongruent findings for noninterfering pain, our results largely suggest that individuals who both work physically demanding jobs and experience activity-interfering pain have low expectations of full-time work past ages 62 or 65. This is consistent with a growing body of research suggesting that pain interference has a significant impact on work ability and early retirement (Hengstebeck et al., 2017; Rice et al., 2011; Van Schaaijk et al., 2020). From this line of prior work, it seems that pain-related limitations are more salient to these outcomes than the presence or intensity of pain. Previous research also suggests that the association between physical work demands and early or disability retirement is strongest among men (Emberland et al., 2017; Falkstedt et al., 2021), though we find physical demands to be predictors of future work expectations for both men and women. However, our finding that physically demanding work is positively associated with low expectations of full-time work after age 62 for men with noninterfering pain, but negatively associated with this expectation for women with noninterfering pain is consistent with previous work finding physically demanding work to be more salient for older men’s labor force participation.

Our findings align with the extant research documenting the negative relationship between physical work demands and perceived “work ability” among people with musculoskeletal pain (Badarin et al., 2022; Oliv et al., 2017; Skovlund et al., 2020). Work ability is defined as a balance between work obligations and cognitive or physical capacity (Ilmarinen, 2009) Low or declining work ability—often due to a combination of health problems and strenuous work conditions—is associated with higher likelihood of taking sick leave and typically precedes early elective or forced retirement due to disability (Boissonneault & De Beer, 2018; Martinez & Fischer, 2019; Prakash et al., 2021). By assessing future work expectations, our study builds upon this prior research and suggests that pain and physical work demands may influence not only current work ability but also one’s assessment of future work ability.

The results of this study should be considered in light of several limitations. First, our analysis is limited to individuals aged 51–56 who were currently employed, which precluded an analysis of individuals who are not working at these ages. Research has shown that individuals who are not consistently employed in their 50s have high rates of disability and health problems and are unlikely to return to paid employment in their 60s (Truesdale et al., 2022). This suggests that some of these individuals may have already left the labor force due to the combination of pain and work demands, and they would likely have low expectations of future work had they been included in our study. Second, the questions about the percent chance of working full-time past ages 62 and 65 do not specifically measure *retirement* expectations. In fact, the questions may also capture intentions to transition to part-time work, which would be consistent with previous research finding that workers with pain are more likely to engage in part-time work (Adams & Salomons, 2021). However, because older workers’ most common response to new health-related work limitations is to stop working (Schimmel Hyde et al., 2022), we argue that our findings speak to expectations about retirement among individuals with physically demanding jobs who experience pain. An additional limitation of these questions is that whereas 62 is widely regarded as an early retirement age, 65 is not necessarily the normal or full retirement eligibility age for all HRS participants. Nevertheless, expectations about working full-time past this age are informative because low expectations of working full-time past age 65 suggest that individuals may be unwilling or unable to continue working until the policy-imposed age at which they may receive full Social Security benefits. Future research should examine actual labor force participation at these ages and whether workers transition to part-time or less physically demanding jobs to accommodate for their pain. Furthermore, because the period of rising pain prevalence coincided with the initial rise of opioid prescriptions and subsequent recognition of an opioid epidemic, future research should assess how the relationship between pain and work expectations may have changed over time (Zajacova et al., 2021a, 2021b). Such continued research will provide additional insights into the implications of increasing pain prevalence among older adults for policies encouraging working longer.

## Conclusion

This study found that among adults in their 50s, the co-occurrence of pain and physical work demands was associated with low expectations of working full-time past ages 62 or 65. Our findings are important in light of the rising pain prevalence among middle-aged adults while individuals are, simultaneously, expected to work longer to receive full retirement benefits (Johnson, 2018; Zajacova et al., 2021a; Zelaya et al., 2020). This work is increasingly pertinent as policymakers continue to propose older Social Security eligibility ages as solutions to improve the system’s solvency (Johnson, 2018). Working longer may be an attractive option for the majority of older workers who are still in good health, but it may not be feasible for all. Some individuals with pain may be able to qualify for SSDI benefits and avoid the financial penalty associated with claiming Social Security benefits early, but individuals with pain often face barriers to SSDI eligibility if their pain is not medically verifiable (Houtenville & Ozabaci, 2019; Maestas, 2019; Zajacova et al., 2021b). To encourage working longer at the population level without disproportionately harming workers in particularly strenuous jobs, some have suggested introducing occupation or industry-specific retirement eligibility ages (Vermeer et al., 2016). An alternative to determining benefits eligibility by age would be to determine eligibility by years of work, which would allow those who started working earlier—frequently those who do not pursue higher education and who work in more physically demanding jobs—to claim at earlier ages without being financially penalized (Johnson, 2018). On the employer side, research has shown that workplace interventions to limit physically strenuous activity and offer more attention to the balance of work and recovery can significantly reduce the amount of time workers take off for sickness, likely due to reduced rates of injury and disability (Oude Hengel et al., 2013; Shiri et al., 2020). Such “age-friendly” workplace interventions can have the added benefit of preventing injuries and musculoskeletal disorders among workers of all ages (Grosch & Scholl, 2020). Future research should continue to explore ways that policies and employers can encourage and facilitate working longer for some without disproportionately harming workers in physically demanding jobs who experience pain.

## Supplementary Material

igad089_suppl_Supplementary_Material
