# Supplementary material for: Pain, Physical Demands at Work, and Future Work Expectations Among Older Adults in the United States
Source: Innov Aging. 2023 Aug 22;7(10):igad089. doi: 10.1093/geroni/igad089 (PMC10714917; doi:10.1093/geroni/igad089)
Supplement: igad089_suppl_Supplementary_Material [file igad089_suppl_supplementary_material.docx]

**Online Supplementary Material**

| A1: Sample selection diagram for analytic sample............................................................... | 2 |
| --- | --- |
| A2: Distribution of self-reported probabilities of working full-time past 62 or 65 by  gender.................................................................................................................................... | 3 |
| A3: Additional information on variables capturing financial preparedness for retirement.............................................................................................................................. | 4 |
| A4: Associations between all variables and expectation of not working full time past age 62: Odds ratios and 95% confidence intervals...................................................................... | 6 |
| A5: Associations between all variables and expectation of not working full time past age 65: Odds ratios and 95% confidence intervals...................................................................... | 8 |
| A6: Associations between pain, high physical effort work, and alternative specifications of expectation of not working full time past ages 62 and 65: Odds ratios and 95% confidence intervals............................................................................................................... | 10 |
| A7: Associations between binary measure of pain, high physical effort work, and expectation of not working full time past ages 62 and 65: Odds ratios and 95% confidence intervals............................................................................................................... | 12 |
| A8: Associations between measure of pain severity, high physical effort work, and expectation of not working full time past ages 62 and 65: Odds ratios and 95% confidence intervals............................................................................................................... | 13 |

**A1: Sample selection diagram.** HRS = Health and Retirement Study. Inconsistent work expectations refer to individuals who report a lower probability of working full-time past age 62 than age 65.

**A2: Distribution of self-reported probabilities of working full-time past 62 or 65 by**

**gender.** HRS respondents who were currently working for pay were asked “Thinking about work in general and not just your present job, what do you think the chances are that you will be working full-time after you reach age 62?” The dashed line represents where we dichotomized this variable; individuals reporting a 25% chance or less expected not to work full time past 62 and 65.

**A3: Additional information on variables capturing financial preparedness for retirement**

In our analyses we include several financial measures as covariates because expectations about working full-time past ages 62 and 65 are likely informed by one’s financial position. These include:

**Presence of a defined benefit plan at one’s current job:** Respondents first report whether they have a pension or pensions at their current job. They are then asked to report the type of pension plan for up to four pensions, with “defined benefit”, “defined contribution”, and “both pension types” as potential responses. We coded individuals who reported a defined benefit pension plan for at least one pension type as having a defined benefit plan at their current job.

Household income and asset variables: We include the following variables as measures of the household’s financial preparedness to retire. For each household, one respondent is designated as the financial respondent who reports about several income and wealth measures at the household level. RAND imputes values for missing data on each of these income and wealth components. Each of these variables is reported in nominal dollars, and for the analyses we have standardized all income and asset values to 1998 dollars to account for inflation; we also log-transformed these variables (adding $1 to each to include individuals with $0 of income or assets) for analysis to adjust for skewed distributions.

**Household income:** We use the RAND-constructed variable for total household income (RAND variable hwitot), which is the sum of all income received by the respondent and spouse, including earnings, pensions and annuities, Supplemental Security Income and Social Security Disability Insurance income, Social Security retirement benefits, unemployment and workers compensation, other government benefits, household capital income, and other income.

**Household non-housing net worth:** We use the RAND-constructed variable for total non-housing household financial wealth (RAND variable hwatotf). This includes the sum of all financial assets (stocks, bonds, bank accounts, etc.) less the sum of all debt. This variable does not include individual retirement account (IRA) balances. We do not consider housing wealth in this analysis because it is not liquid and easily able to replace earned income upon retirement. For individuals with a negative net worth, we have coded household non-housing net worth to have a floor of $0.

**Household debt**: For individuals with a negative net worth on the above household non-housing net wealth variable, we coded their net worth as $0 and have created a separate debt variable to capture the amount of debt.

**Household total individual retirement (IRA) account balances:** We use the RAND-constructed variable for the net value of the household’s IRA and Keogh accounts (RAND variable hwAIRA).

**Household total defined contribution account balances:** Each HRS respondent reports the amount in each defined contribution (DC) pension plan account at the current employer. If individuals do not report having a pension at their current job, or report having a pension that is not a DC plan, we set their DC balance to 0. If they report having a pension, but either the type or amount is missing, we set their DC balance to missing so it can be imputed. For individuals without a spouse, the spouse’s DC balance is set to 0. For individuals with a spouse whose pension information is missing, we set the spousal DC balance to missing so it can be imputed. We sum the respondent and spouse DC balance for a total household DC balance.

While these variables are all related to household financial position, they are only modestly correlated with one another, as displayed in Table A3.

**Table A3: Correlations between household financial variables**

|  | (1) Income | (2)  Wealth | (3)  Debt | (4)  Defined contribution plan balance | (5)  IRA balance |
| --- | --- | --- | --- | --- | --- |
| (1) Income | 1.00 | 0.34 | -0.07 | 0.27 | 0.30 |
| (2) Wealth |  | 1.00 | -0.70 | 0.20 | 0.41 |
| (3) Debt |  |  | 1.00 | -0.05 | -0.17 |
| (4) Defined  contribution plan balance |  |  |  | 1.00 | 0.19 |
| (5) IRA balance |  |  |  |  | 1.00 |

Note: These variables have been indexed to 1998 dollars to account for inflation and logged to account for the skewed distribution of the variables in the original scale

**A4: Associations between all variables and expectation of not working full time past age 62: Odds ratios and 95% confidence intervals**

|  | **Men** | | **Women** | |
| --- | --- | --- | --- | --- |
|  | **Model 1** | **Model 2** | **Model 1** | **Model 2** |
| Pain interference  (ref = no pain) |  |  |  |  |
| Non-interfering pain | 1.08  (0.99, 1.17) | 0.93  (0.84, 1.03) | 1.02  (0.95, 1.11) | **1.12**  (1.02, 1.22) |
| Interfering pain | **1.19**  (1.09, 1.30) | **1.20**  (1.08, 1.33) | **1.21**  (1.13, 1.30) | **1.16**  (1.07, 1.25) |
| High physical effort job | **1.19**  (1.10, 1.27) | **1.10**  (1.01, 1.19) | **1.13**  (1.06, 1.22) | **1.15**  (1.06, 1.25) |
| High physical effort job X  non-interfering pain |  | **1.62**  (1.35, 1.93) |  | 0.69  (0.56, 0.85) |
| High physical effort job X interfering pain |  | 1.01  (0.84, 1.21) |  | 1.18  (1.00, 1.39) |
| Age (centered on 54) | **0.94** (0.93, 0.96) | **0.94** (0.93, 0.96) | **0.96** (0.95, 0.98) | **0.96** (0.95, 0.97) |
| Some college | **1.62** (1.46, 1.79) | **1.61** (1.46, 1.79) | 0.93 (0.85, 1.01) | 0.93 (0.85, 1.01) |
| High school or equivalent | **1.67** (1.54, 1.81) | **1.67** (1.54, 1.81) | 1.03 (0.96, 1.11) | 1.03 (0.96, 1.11) |
| Less than high school | **2.06** (1.81, 2.34) | **2.06** (1.81, 2.35) | **1.71** (1.51, 1.94) | **1.71** (1.50, 1.94) |
| Black | **2.02** (1.83, 2.23) | **2.02** (1.83, 2.23) | **1.98** (1.81, 2.16) | **1.98** (1.81, 2.16) |
| Hispanic | **1.61** (1.43, 1.81) | **1.61** (1.43, 1.81) | **1.55** (1.39, 1.73) | **1.55** (1.39, 1.73) |
| Other | 0.90 (0.79, 1.03) | **0.88** (0.77, 1.01) | **1.54** (1.37, 1.74) | **1.55** (1.37, 1.75) |
| Separated/divorced | **0.75** (0.69, 0.82) | **0.75** (0.69, 0.82) | **0.67** (0.62, 0.73) | **0.68** (0.63, 0.73) |
| Widowed | **0.66** (0.50, 0.87) | **0.66** (0.50, 0.87) | **0.80** (0.70, 0.91) | **0.79** (0.70, 0.90) |
| Never married | **1.14** (1.02, 1.28) | **1.12** (1.00, 1.26) | **0.68** (0.61, 0.76) | **0.69** (0.61, 0.77) |
| Full retirement age (centered on 66) | **0.72** (0.67, 0.78) | **0.72** (0.66, 0.78) | **0.53** (0.50, 0.57) | **0.53** (0.50, 0.57) |
| Foreign-born | **1.15** (1.03, 1.28) | **1.17** (1.05, 1.3) | 0.95 (0.86, 1.05) | 0.95 (0.86, 1.05) |
| High stress job | **1.22** (1.14, 1.3) | **1.22** (1.14, 1.3) | **1.07** (1.01, 1.14) | **1.07** (1.00, 1.14) |
| Total years worked (centered on 26) | **1.02** (1.02, 1.03) | **1.02** (1.02, 1.03) | 1.00 (1.00, 1.00) | 1.00 (1.00, 1.00) |
| Self-employed | **0.78** (0.71, 0.85) | **0.78** (0.71, 0.85) | **0.75** (0.69, 0.81) | **0.75** (0.69, 0.81) |
| Belongs to a union | **1.65** (1.54, 1.78) | **1.66** (1.54, 1.78) | **1.47** (1.37, 1.57) | **1.47** (1.37, 1.57) |
| Service | 0.89 (0.80, 1.00) | 0.89 (0.80, 1.00) | **1.31** (1.20, 1.43) | **1.32** (1.20, 1.44) |
| Sales/clerical | **0.84** (0.77, 0.92) | **0.84** (0.76, 0.92) | 1.07 (1.00, 1.14) | 1.07 (1.00, 1.14) |
| Manual | **0.91** (0.84, 0.99) | **0.91** (0.83, 0.99) | **1.16** (1.04, 1.29) | **1.16** (1.04, 1.29) |
| Part-time | **3.91** (3.48, 4.40) | **3.91** (3.48, 4.40) | **3.45** (3.16, 3.77) | **3.45** (3.17, 3.77) |
| More than full-time | **0.85** (0.79, 0.93) | **0.85** (0.79, 0.92) | **0.84** (0.75, 0.94) | **0.84** (0.75, 0.93) |
| Log income | **0.93** (0.91, 0.95) | **0.93** (0.91, 0.95) | 1.02 (1.00, 1.05) | 1.03 (1.00, 1.05) |
| Log non-housing financial wealth^a^ | **1.06** (1.05, 1.07) | **1.06** (1.05, 1.07) | **1.04** (1.03, 1.05) | **1.04** (1.03, 1.05) |
| Log non-mortgage debt^a^ | **1.02** (1, 1.03) | **1.02** (1.01, 1.03) | **0.99** (0.98, 1.00) | **0.99** (0.98, 1.00) |
| Log defined contribution plan balance | **1.02** (1.01, 1.03) | **1.02** (1.01, 1.03) | **1.02** (1.01, 1.03) | **1.02** (1.01, 1.03) |
| Log IRA balance^a^ | 1.01 (1.00, 1.01) | 1.00 (1.00, 1.01) | **1.01** (1.00, 1.01) | **1.01** (1.00, 1.01) |
| Defined benefit plan | **2.20** (2.05, 2.36) | **2.19** (2.04, 2.35) | **1.40** (1.31, 1.49) | **1.40** (1.31, 1.49) |
| Former smoker | **1.13** (1.06, 1.21) | **1.13** (1.06, 1.2) | **0.94** (0.89, 1.00) | **0.94** (0.89, 0.99) |
| Current smoker | **1.16** (1.07, 1.25) | **1.15** (1.07, 1.25) | 0.94 (0.88, 1.01) | 0.94 (0.88, 1.01) |
| Obese | **1.07** (1.00, 1.14) | **1.07** (1.00, 1.14) | **0.93** (0.86, 1.00) | 0.93 (0.87, 1.00) |
| Count of chronic conditions | **1.13** (1.08, 1.17) | **1.13** (1.08, 1.17) | 1.02 (0.99, 1.06) | 1.02 (0.99, 1.06) |
| Three or more depressive symptoms | **0.91** (0.83, 0.99) | **0.90** (0.83, 0.98) | **1.11** (1.04, 1.19) | **1.11** (1.04, 1.19) |
| Number of respondents | 4,691 | 4,691 | 5,667 | 5,667 |

Bold values indicate significance at p<0.05. Results are pooled across 10 multiply imputed data sets.

**A5: Associations between all variables and expectation of not working full time past age 65: Odds ratios and 95% confidence intervals**

|  | **Men** | | **Women** | |
| --- | --- | --- | --- | --- |
|  | **Model 3** | **Model 4** | **Model 3** | **Model 4** |
| Pain interference  (ref = no pain) |  |  |  |  |
| Non-interfering pain | **1.20**  (1.11, 1.29) | **1.20**  (1.10, 1.31) | 0.95  (0.88, 1.03) | 0.99  (0.91, 1.08) |
| Interfering pain | **1.15**  (1.06, 1.25) | 1.08  (0.99, 1.18) | **1.12**  (1.05, 1.20) | **1.09**  (1.01, 1.18) |
| High physical effort job | **1.05**  (0.98, 1.12) | 1.01  (0.93, 1.09) | **1.18**  (1.11, 1.26) | **1.19**  (1.09, 1.29) |
| High physical effort job X  non-interfering pain |  | 1.01  (0.85, 1.18) |  | 0.83  (0.68, 1.03) |
| High physical effort job X interfering pain |  | **1.25**  (1.06, 1.47) |  | 1.10  (0.93, 1.31) |
| Age (centered on 54) | 0.99 (0.98, 1.01) | 0.99 (0.98, 1.01) | 0.99 (0.97, 1.00) | 0.99 (0.97, 1.00) |
| Some college | **1.47** (1.34, 1.61) | **1.47** (1.34, 1.61) | 0.96 (0.89, 1.04) | 0.96 (0.89, 1.05) |
| High school or equivalent | **1.57** (1.46, 1.7) | **1.58** (1.46, 1.7) | **1.12** (1.05, 1.2) | **1.12** (1.05, 1.20) |
| Less than high school | **1.84** (1.62, 2.09) | **1.85** (1.63, 2.1) | **1.65** (1.45, 1.87) | **1.65** (1.45, 1.87) |
| Black | **1.93** (1.76, 2.11) | **1.94** (1.76, 2.12) | **1.93** (1.77, 2.1) | **1.93** (1.77, 2.10) |
| Hispanic | **1.48** (1.33, 1.64) | **1.48** (1.33, 1.65) | **1.40** (1.26, 1.55) | **1.40** (1.26, 1.56) |
| Other | **0.80** (0.71, 0.90) | **0.80** (0.71, 0.9) | **1.23** (1.09, 1.38) | **1.23** (1.09, 1.38) |
| Separated/divorced | 0.93 (0.86, 1.01) | 0.93 (0.86, 1.01) | **0.64** (0.60, 0.69) | **0.65** (0.60, 0.69) |
| Widowed | **0.67** (0.52, 0.86) | **0.67** (0.52, 0.86) | 0.90 (0.79, 1.02) | 0.90 (0.79, 1.02) |
| Never married | 0.94 (0.84, 1.05) | 0.94 (0.84, 1.05) | **0.62** (0.55, 0.68) | **0.62** (0.56, 0.69) |
| Full retirement age (centered on 66) | **0.73** (0.68, 0.79) | **0.73** (0.68, 0.79) | **0.64** (0.61, 0.69) | **0.64** (0.6, 0.68) |
| Foreign-born | **1.36** (1.24, 1.50) | **1.36** (1.24, 1.5) | 1.09 (0.99, 1.21) | 1.09 (0.99, 1.21) |
| High stress job | **1.18** (1.11, 1.25) | **1.17** (1.11, 1.24) | **1.15** (1.08, 1.21) | **1.14** (1.08, 1.21) |
| Total years worked (centered on 26) | **1.02** (1.02, 1.02) | **1.02** (1.02, 1.02) | **1.00** (1.00, 1.01) | **1.00** (1.00, 1.01) |
| Self-employed | **0.61** (0.56, 0.66) | **0.61** (0.56, 0.66) | **0.71** (0.65, 0.76) | **0.71** (0.65, 0.76) |
| Belongs to a union | **1.86** (1.73, 1.99) | **1.85** (1.73, 1.98) | **1.51** (1.41, 1.62) | **1.51** (1.41, 1.62) |
| Service | 1.10 (0.99, 1.22) | 1.10 (1.00, 1.22) | **1.51** (1.39, 1.64) | **1.51** (1.39, 1.65) |
| Sales/clerical | 0.93 (0.86, 1.01) | 0.93 (0.86, 1.01) | **1.07** (1.00, 1.13) | **1.07** (1.00, 1.14) |
| Manual | 1.06 (0.99, 1.15) | 1.07 (0.99, 1.15) | **1.28** (1.16, 1.42) | **1.28** (1.16, 1.42) |
| Part-time | **2.15** (1.93, 2.39) | **2.15** (1.94, 2.39) | **2.39** (2.20, 2.59) | **2.39** (2.20 2.59) |
| More than full-time | **0.76** (0.71, 0.81) | **0.76** (0.71, 0.81) | **0.77** (0.70, 0.85) | **0.77** (0.70, 0.85) |
| Log income | 0.99 (0.97, 1.01) | 0.99 (0.97, 1.01) | 0.98 (0.96, 1.00) | 0.98 (0.96, 1.00) |
| Log non-housing financial wealth^a^ | **1.06** (1.05, 1.07) | **1.06** (1.05, 1.07) | **1.04** (1.03, 1.05) | **1.04** (1.03, 1.05) |
| Log non-mortgage debt^a^ | **1.02** (1.01, 1.03) | **1.02** (1.01, 1.03) | **0.99** (0.98, 1.00) | **0.99** (0.98, 1.00) |
| Log defined contribution plan balance | **1.03** (1.02, 1.04) | **1.03** (1.02, 1.04) | **1.03** (1.02, 1.04) | **1.03** (1.02, 1.04) |
| Log IRA balance^a^ | **1.02** (1.01, 1.02) | **1.02** (1.01, 1.02) | **1.02** (1.01, 1.02) | **1.02** (1.01, 1.02) |
| Defined benefit plan | **1.89** (1.76, 2.02) | **1.89** (1.77, 2.03) | **1.74** (1.61, 1.88) | **1.74** (1.61, 1.88) |
| Former smoker | 0.97 (0.92, 1.02) | 0.97 (0.92, 1.02) | **0.89** (0.84, 0.94) | **0.89** (0.84, 0.94) |
| Current smoker | **1.30** (1.21, 1.39) | **1.30** (1.21, 1.39) | **0.86** (0.81, 0.92) | **0.86** (0.81, 0.92) |
| Obese | 1.05 (0.99, 1.11) | 1.05 (0.99, 1.11) | 0.97 (0.91, 1.03) | 0.97 (0.91, 1.03) |
| Count of chronic conditions | **1.08** (1.05, 1.12) | **1.08** (1.05, 1.12) | **1.08** (1.04, 1.11) | **1.08** (1.04, 1.11) |
| Three or more depressive symptoms | **1.10** (1.02, 1.18) | **1.10** (1.02, 1.18) | 1.01 (0.95, 1.08) | 1.01 (0.94, 1.07) |
| Number of respondents | 4,691 | 4,691 | 5,667 | 5,667 |

Bold values indicate significance at p<0.05. Results are pooled across 10 multiply imputed data sets.

**A6: Associations between pain, high physical effort work, and alternative specifications of expectation of not working full time past ages 62 and 65: Odds ratios and 95% confidence intervals**

|  | **Men (N = 4,691)** | | **Women (N = 5,667)** | |  |  |
| --- | --- | --- | --- | --- | --- | --- |
| **A) 0% chance of working full time past age 62** | | |  | |  |  |
|  | **Model 1** | **Model 2** | **Model 1** | **Model 2** |  |  |
| Pain interference  (ref = no pain) |  |  |  |  |  |  |
| Non-interfering pain | **1.16** (1.04, 1.29) | 0.97 (0.84, 1.11) | **0.90** (0.81, 1.00) | 1.00 (0.89, 1.12) |  |  |
| Interfering pain | **1.20** (1.07, 1.34) | **1.26** (1.10, 1.43) | **1.32** (1.21, 1.44) | **1.27** (1.16, 1.41) |  |  |
| High physical effort job | **1.24** (1.13, 1.36) | **1.16** (1.04, 1.30) | **1.10** (1.01, 1.20) | **1.12** (1.01, 1.25) |  |  |
| High physical effort job X  non-interfering pain |  | **1.65** (1.32, 2.06) |  | **0.65** (0.50, 0.84) |  |  |
| High physical effort job X interfering pain |  | 0.88 (0.69, 1.11) |  | 1.12 (0.93, 1.36) |  |  |
| **B) 0% chance of working full time past age 65** | | |  | |  |  |
|  | **Model 3** | **Model 4** | **Model 3** | **Model 4** |  |  |
| Pain interference  (ref = no pain) |  |  |  |  |  |  |
| Non-interfering pain | 1.07 (0.99, 1.17) | 1.01 (0.91, 1.11) | **0.84** (0.77, 0.91) | **0.89** (0.81, 0.98) |  |  |
| Interfering pain | 1.06 (0.97, 1.16) | 1.11 (1.00, 1.23) | **1.16** (1.08, 1.25) | **1.18** (1.09, 1.28) |  |  |
| High physical effort job | **1.34** (1.25, 1.44) | **1.33** (1.22, 1.44) | **1.14** (1.07, 1.23) | **1.21** (1.11, 1.32) |  |  |
| High physical effort job X  non-interfering pain |  | **1.21** (1.02, 1.45) |  | **0.75** (0.61, 0.92) |  |  |
| High physical effort job X interfering pain |  | 0.87 (0.72, 1.05) |  | 0.92 (0.78, 1.08) |  |  |
| **C) Up to 50% chance of working full time past age 62** | | | | | |  |
|  | **Model 1** | **Model 2** | **Model 1** | **Model 2** |  |  |
| Pain interference  (ref = no pain) |  |  |  |  |  |  |
| Non-interfering pain | **1.27** (1.18, 1.37) | **1.20** (1.10, 1.31) | 1.05 (0.97, 1.13) | 1.00 (0.92, 1.09) |  |  |
| Interfering pain | **1.27** (1.18, 1.38) | **1.30** (1.19, 1.43) | **1.32** (1.23, 1.41) | **1.32** (1.22, 1.42) |  |  |
| High physical effort job | **1.11** (1.04, 1.18) | **1.08** (1.00, 1.17) | **1.43** (1.34, 1.53) | **1.38** (1.27, 1.5) |  |  |
| High physical effort job X  non-interfering pain |  | **1.23** (1.04, 1.44) |  | **1.24** (1.02, 1.49) |  |  |
| High physical effort job X interfering pain |  | 0.94 (0.80, 1.11) |  | 1.02 (0.87, 1.20) |  |  |
| **D) Up to 50% chance of working full time past age 65** | | | | | | |
|  | **Model 3** | **Model 4** | **Model 3** | **Model 4** |  |  |
| Pain interference  (ref = no pain) |  |  |  |  |  |  |
| Non-interfering pain | **1.25** (1.16, 1.36) | **1.28** (1.17, 1.41) | **1.15** (1.05, 1.25) | **1.12** (1.01, 1.23) |  |  |
| Interfering pain | **1.19** (1.09, 1.29) | **1.25** (1.13, 1.39) | 0.98 (0.90, 1.05) | **0.88** (0.81, 0.96) |  |  |
| High physical effort job | **0.90** (0.84, 0.96) | 0.94 (0.86, 1.02) | **1.24** (1.15, 1.34) | 1.09 (0.99, 1.20) |  |  |
| High physical effort job X  non-interfering pain |  | 0.91 (0.76, 1.09) |  | 1.17 (0.94, 1.46) |  |  |
| High physical effort job X interfering pain |  | **0.83** (0.69, 1.00) |  | **1.59** (1.32, 1.91) |  |  |

Bold values indicate significance at p<0.05. Results are pooled across 10 multiply imputed data sets. All models contain additional controls including age, race/ethnicity, foreign-born status, marital status, educational attainment, the broad category of the respondent’s main occupation, an indicator for whether the respondent is self-employed, an indicator for whether the respondent belongs to a union, an indicator for whether the respondent’s current job involves a lot of stress, the typical weekly work schedule, smoking status, an indicator for whether the respondent is obese, an indicator for whether the respondent scores 3 or higher on the CESD scale, the count of chronic conditions, whether the respondent has a defined benefit retirement plan, log household income from all sources, log household non-housing net worth, log household debt, log household IRA balances, log household defined contribution account balances, the total number of years worked, and the age at which respondents will become eligible for full retirement benefits.

**A7: Associations between binary measure of pain, high physical effort work, and expectation of not working full time past ages 62 and 65: Odds ratios and 95% confidence intervals**

| **A) Expecting not to work full time past age 62** | | |  | |
| --- | --- | --- | --- | --- |
|  | **Men** | | **Women** | |
|  | **Model 1** | **Model 2** | **Model 1** | **Model 2** |
| Pain | **1.13**  (1.06, 1.21) | 1.05  (0.98, 1.14) | **1.13**  (1.06, 1.19) | **1.13**  (1.06, 1.21) |
| High physical effort job | **1.18**  (1.10, 1.27) | **1.09**  (1.00, 1.19) | **1.14**  (1.06, 1.22) | **1.15**  (1.06, 1.25) |
| High physical effort job X pain |  | **1.28**  (1.11, 1.47) |  | 0.96  (0.84, 1.11) |
| Number of respondents | 4,691 | 4,691 | 5,667 | 5,667 |
| **B) Expecting not to work full time past age 65** | | |  | |
|  | **Men** | | **Women** | |
|  | **Model 3** | **Model 4** | **Model 3** | **Model 4** |
| Pain | **1.18**  (1.11, 1.25) | **1.14**  (1.07, 1.22) | 1.04  (0.99, 1.10) | 1.05  (0.98, 1.11) |
| High physical effort job | 1.05  (0.98, 1.12) | 1.01  (0.94, 1.09) | **1.18**  (1.11, 1.26) | **1.19**  (1.09, 1.29) |
| High physical effort job X pain |  | 1.12  (0.98, 1.27) |  | 0.99  (0.86, 1.14) |
| Number of respondents | 4,691 | 4,691 | 5,667 | 5,667 |

Bold values indicate significance at p<0.05. Results are pooled across 10 multiply imputed data sets. All models contain additional controls including age, race/ethnicity, foreign-born status, marital status, educational attainment, the broad category of the respondent’s main occupation, an indicator for whether the respondent is self-employed, an indicator for whether the respondent belongs to a union, an indicator for whether the respondent’s current job involves a lot of stress, the typical weekly work schedule, smoking status, an indicator for whether the respondent is obese, an indicator for whether the respondent scores 3 or higher on the CESD scale, the count of chronic conditions, whether the respondent has a defined benefit retirement plan, log household income from all sources, log household non-housing net worth, log household debt, log household IRA balances, log household defined contribution account balances, the total number of years worked, and the age at which respondents will become eligible for full retirement benefits.

**A8: Associations between measure of pain severity, high physical effort work, and expectation of not working full time past ages 62 and 65: Odds ratios and 95% confidence intervals**

| **A) Expecting not to work full time past age 62** | | |  | |
| --- | --- | --- | --- | --- |
|  | **Men** | | **Women** | |
|  | **Model 1** | **Model 2** | **Model 1** | **Model 2** |
| Pain severity (ref = no pain) |  |  |  |  |
| Mild pain | **1.13**  (1.04, 1.24) | 1.01  (0.91, 1.12) | **1.16**  (1.07, 1.26) | **1.25**  (1.14, 1.37) |
| Moderate/severe pain | **1.13**  (1.04, 1.23) | 1.10  (0.99, 1.21) | **1.10**  (1.03, 1.18) | 1.06  (0.98, 1.15) |
| High physical effort job | **1.18**  (1.1, 1.27) | **1.09**  (1.00, 1.19) | **1.14**  (1.06, 1.22) | **1.15**  (1.06, 1.25) |
| High physical effort job X  mild pain |  | **1.53**  (1.26, 1.85) |  | **0.71**  (0.57, 0.89) |
| High physical effort job X  moderate/severe pain |  | 1.12  (0.94, 1.34) |  | 1.12  (0.95, 1.32) |
| Number of respondents | 4,691 | 4,691 | 5,667 | 5,667 |
| **B) Expecting not to work full time past age 65** | | |  | |
|  | **Men** | | **Women** | |
|  | **Model 3** | **Model 4** | **Model 3** | **Model 4** |
| Pain severity (ref = no pain) |  |  |  |  |
| Mild pain | **1.25**  (1.15, 1.35) | **1.29**  (1.18, 1.42) | 1.00 (0.92, 1.09) | 1.04  (0.95, 1.14) |
| Moderate/severe pain | **1.12**  (1.04, 1.21) | 1.02  (0.93, 1.12) | 1.07  (1.00, 1.15) | 1.05  (0.97, 1.13) |
| High physical effort job | 1.05  (0.98, 1.12) | 1.01  (0.93, 1.09) | **1.18**  (1.11, 1.26) | **1.19**  (1.09, 1.29) |
| High physical effort job X  mild pain |  | 0.87  (0.73, 1.04) |  | 0.83  (0.67, 1.02) |
| High physical effort job X  moderate/severe pain |  | **1.35**  (1.15, 1.57) |  | 1.07  (0.91, 1.26) |
| Number of respondents | 4,691 | 4,691 | 5,667 | 5,667 |

Bold values indicate significance at p<0.05. Results are pooled across 10 multiply imputed data sets. All models contain additional controls including age, race/ethnicity, foreign-born status, marital status, educational attainment, the broad category of the respondent’s main occupation, an indicator for whether the respondent is self-employed, an indicator for whether the respondent belongs to a union, an indicator for whether the respondent’s current job involves a lot of stress, the typical weekly work schedule, smoking status, an indicator for whether the respondent is obese, an indicator for whether the respondent scores 3 or higher on the CESD scale, the count of chronic conditions, whether the respondent has a defined benefit retirement plan, log household income from all sources, log household non-housing net worth, log household debt, log household IRA balances, log household defined contribution account balances, the total number of years worked, and the age at which respondents will become eligible for full retirement benefits.
